# Intensive lipid-lowering therapy for early achievement of guideline-recommended LDL-cholesterol levels in patients with ST-elevation myocardial infarction (“Jena auf Ziel”)

**DOI:** 10.1007/s00392-022-02147-3

**Published:** 2023-01-05

**Authors:** Umidakhon Makhmudova, Beasat Samadifar, Aurel Maloku, Pellumb Haxhikadrija, Jens-Arndt Geiling, Robert Römer, Bernward Lauer, Sven Möbius-Winkler, Sylvia Otto, P. Christian Schulze, Oliver Weingärtner

**Affiliations:** Division of Cardiology, Department of Internal Medicine I, University Hospital Jena, Am Klinikum 1, 07747 Jena, Germany

**Keywords:** LDL cholesterol, Secondary prevention, ST-elevation myocardial infarction, LDL cholesterol target attainment

## Abstract

**Background and aims:**

Currently, less than 20% of patients at very high-risk achieve ESC/EAS dyslipidemia guideline-recommended LDL-C target levels in Europe. “Jena auf Ziel—JaZ” is a prospective cohort study in which early combination therapy with atorvastatin 80 mg and ezetimibe 10 mg was initiated on admission in patients with ST-elevation myocardial infarction (STEMI) and lipid-lowering therapy was escalated during follow-up with bempedoic acid and PCSK9 inhibitors to achieve recommended LDL-C targets in all patients. Moreover, we evaluated side-effects of lipid-lowering therapy.

**Methods:**

Patients admitted with STEMI at Jena University Hospital were started on atorvastatin 80 mg and ezetimibe 10 mg on admission. Patients were followed for EAS/ESC LDL-C target achievement during follow-up.

**Results:**

A total of 85 consecutive patients were enrolled in the study. On discharge, 32.9% achieved LDL-C targets on atorvastatin 80 mg and ezetimibe 10 mg. After 4–6 weeks, 80% of all patients on atorvastatin 80 mg and ezetimibe started at the index event were on ESC/EAS LDL-C targets. In 20%, combined lipid-lowering therapy was escalated with either bempedoic acid or PCSK9 inhibitors. All patients achieved LDL-C levels of or below 55 mg/dL during follow-up on triple lipid-lowering therapy. Combined lipid-lowering therapy was well-tolerated with rare side effects.

**Conclusions:**

Early combination therapy with a high-intensity statin and ezetimibe and escalation of lipid-lowering therapy with either bempedoic acid or PCSK9 inhibitors gets potentially all patients with STEMI on recommended ESC/EAS LDL-C targets without significant side effects.

**Graphical abstract:**

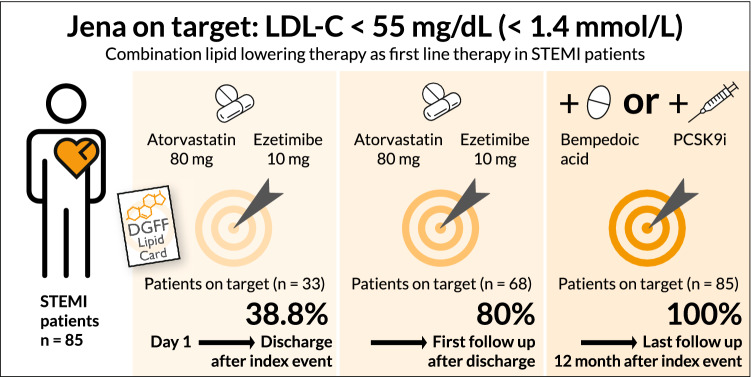

**Supplementary Information:**

The online version contains supplementary material available at 10.1007/s00392-022-02147-3.

## Introduction

Elevated low-density lipoprotein (LDL-C) cholesterol concentrations are a causal risk factor for atherosclerotic cardiovascular disease [1]. The European Society of Cardiology (ESC) and the European Atherosclerosis Society (EAS) released updated guidelines for the management of dyslipidemias in 2020 [2]. For patients with a recent myocardial infarction (MI), the guidelines recommend achieving an LDL-C level of < 1.4 mmol/L (< 55 mg/dL) (Class 1, Level A) [2]. To reach the LDL-C target, lifestyle modifications and treatment with high-intensity statins are recommended. If the target is not reached after 4–6 weeks despite lifestyle modification and maximally tolerated statin therapy, add-on therapy with ezetimibe (Class 1, Level B) and thereafter a proprotein convertase subtilisin/kexin type 9 (PCSK9) inhibitor (Class 1, level A) is recommended [2]. The 2020 guidelines present a lower LDL-C goal and recommend more aggressive LDL-C lowering therapy for patients with an MI, compared with the 2016 ESC/EAS guidelines [2, 3]. The nationwide SWEDEHEART registry demonstrated that only 17.1% of the 25,466 patients included achieved the newly set LDL-C target and 82.9% of the patients would be eligible for expanded lipid-lowering therapy as they had not attained the target of an LDL-C level of < 1.4 mmol/L [4]. In a Monte Carlo simulation model, 92% reached LDL-C targets when the use of high-intensity statins, ezetimibe, and PCSK9 inhibitors was maximized. Furthermore, a follow-up for mortality and major cardiovascular events of SWEDEHEART revealed that a reduction of 2.0 mmol/L (80 mg/dL) in LDL-C 4–6 weeks after an MI was associated with a dramatic reduction in all-cause mortality and major cardiovascular events of up to 70% over a follow-up period of 3.7 years [5]. On this background, data from the EU-Wide Cross-Sectional Observational Study of Lipid-Modifying Therapy Use in Secondary and Primary Care (DaVinci) and Getting to an Improved Understanding of Low-Density Lipoprotein Cholesterol and Dyslipidemia Management (GOULD) registers are sobering as less than 20% of high-risk patients currently achieve LDL-C targets in Europe and the US. This pinpoints a need for improved preventive strategies [6, 7]. Data from an online survey among cardiologists in seven European countries revealed that 78% of patients with acute coronary syndrome (ACS) were not at goal (< 1.8 mmol/L, according to 2016 ESC/EAS guidelines) at the first post-discharge follow-up and in only 41% lipid-lowering therapy was escalated [8]. As a result, 68% and 62% still did not achieve recommended LDL-C targets at the second and third follow-up visits, respectively [8].

The use of combined lipid-lowering therapy with a high-intensity statin and the cholesterol absorption inhibitor ezetimibe early has several advantages and is an effective way of lowering LDL-C [9–13].

“Jena auf Ziel” (JaZ) is a prospective cohort study aiming to achieve EAS/ESC LDL-C targets in patients admitted with ST-elevation myocardial infarction (STEMI). On the day of admission, combined lipid-lowering therapy (atorvastatin 80 mg, ezetimibe 10 mg) was initiated and escalated with either bempedoic acid or PCSK9 inhibitors if LDL-C targets were not reached during the time to the first follow-up after the index event.

## Methods

**Fig. 1:**
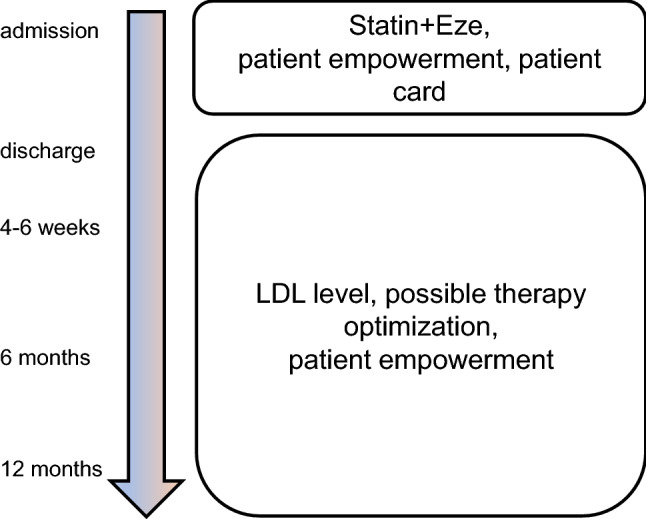
JaZ—study flow. Patients started on atorvastatin 80 mg/ezetimibe 10 mg combination therapy on admission. LDL-C is measured on admission, on discharge, after 6–8 weeks and during further follow-ups. Patient empowerment is used to increase therapy adherence

“Jena auf Ziel—JaZ” is a prospective cohort study in which patients admitted to the Jena University Hospital with STEMI from January 1st to December 31st, 2021, were included. The study was approved by the Local Ethics Committee (5219-07/17). Combined lipid-lowering therapy with a high-intensity statin (atorvastatin 80 mg) and the cholesterol absorption inhibitor ezetimibe (10 mg) was started on admission. During the hospital stay, patients were educated about cardiovascular risk modification, particularly about cholesterol as a significant cardiovascular risk factor, and therapeutic options to reach EAS/ESC-LDL-C targets for very high-risk patients. LDL-C levels were assessed upon admission, during the hospital stay, and discharge (Fig. 1A). The lipid profile was documented on patient cards. The primary outcome was LDL-C target achievement (LDL-C < 1.4 mmol/L, or < 55 mg/dL). If the EAS/ESC targets were not reached, lipid-lowering therapy was escalated with either bempedoic acid or PCSK9 inhibitors (Fig. 1).

## Results

### Baseline characteristics

**Fig. 2:**
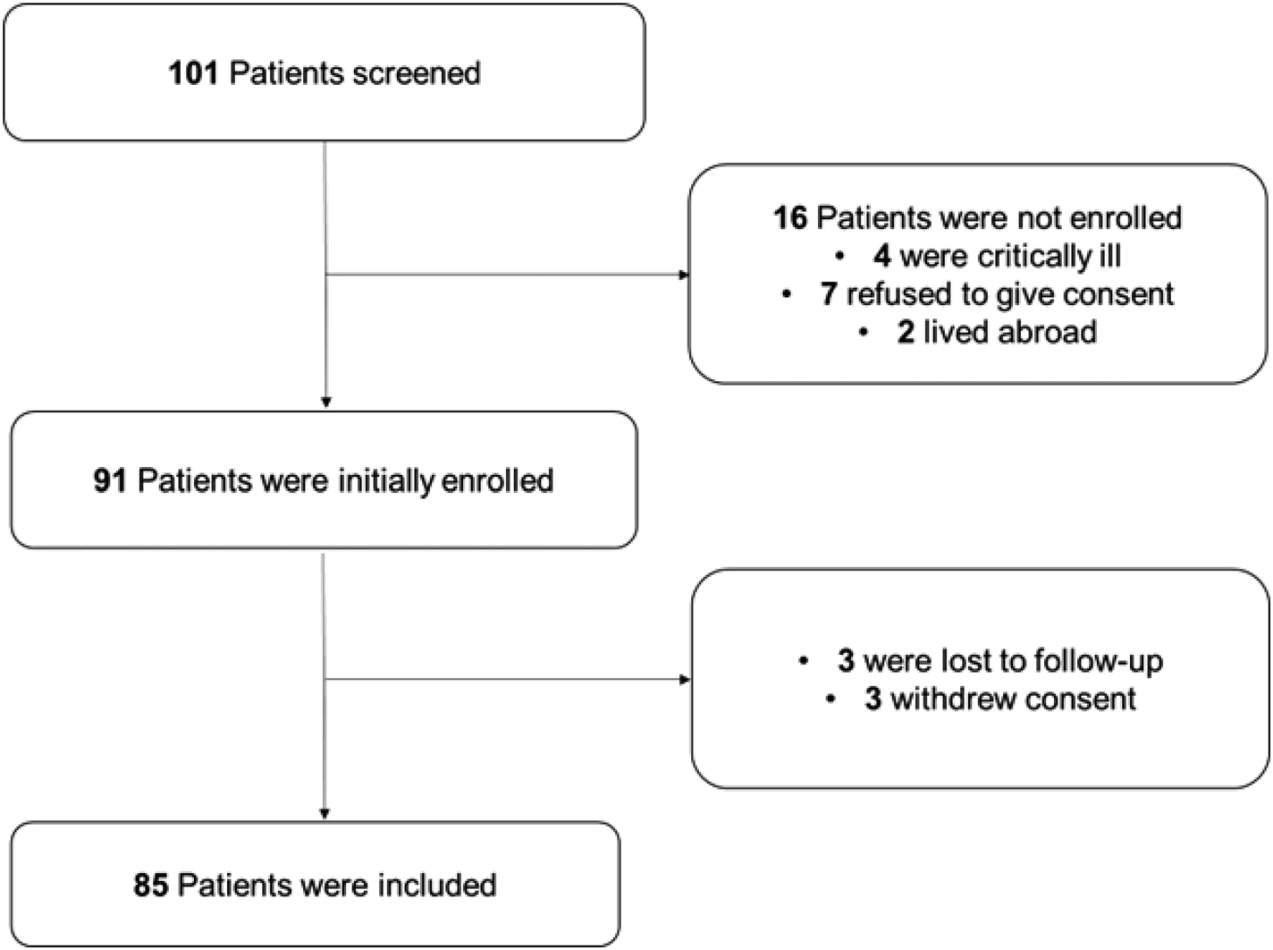
Study diagram in accordance with the STROBE checklist

Ninety-one patients admitted with STEMI to Jena University Hospital were consecutively enrolled in this observational study between January 1st and December 31st, 2021. Three patients refused to participate in the study after inclusion, and three were lost to follow-up. Thus, a total of 85 patients were included in this analysis (Fig. 2). Of 85 included patients, 14.1% were female. Patients were 65 (IQR 53–74) years old. Baseline characteristics are shown in Table 1.

**Table 1:** Baseline characteristics of patients (*n* = 85)

Variable	Value
Demographic and clinical parameters	
Age, years (median, IQR)	65 (53–74)
Women, *n* (%)	12 (14.1)
Smokers, *n* (%)	
Current	26 (30.6)
Former	7 (8.2)
BMI, kg/m^2^ (mean, SD)	27.7 (4.6)
RR syst., mmHg (mean, SD)	145.6 (27.7)
Lipid-lowering therapy at baseline, *n* (%)	
High-intensity statin	3 (3.5)
Moderate-intensity statin	7 (8.2)
Low-intensity statin	2 (2.4)
High-intensity statin + ezetimibe	1 (1.2)
High-intensity statin + ezetimibe + PCSK9mab	1 (1.2)
Laboratory values	
Total cholesterol, mmol/L (mean, SD)	4.6 (1.2)
LDL-C, mmol/L (mean, SD)	3.2 (1.2)
HDL-C, mmol/L (mean, SD)	1.2 (0.3)
TG, mmol/L (mean, SD)	1.3 (1.3)
Lipoprotein (a), nmol/L (median, IQR)	21.0 (20.0–67.5)
Troponin, pg/ml (median, IQR)	3234.0 (1013.0–6830.2)
HbA1C, % (mean, SD)	5.9 (0.8)
eGFR, ml/Min (median, IQR)	80.8 (68.0–91.3)
Comorbidities, *n* (%)	
Diabetes	13 (15.3)
Hypertension	54 (63.5)

Continuous variables are shown as median (IQR) or mean (SD), categorical variables are *n* (%). For lipids: multiply by 38.66976 to convert mmol/L to mg/dL

### LDL-C target attainment

The average LDL-C on admission was 3.2 ± 1.2 mmol/L (123.4 ± 44.9 mg/dL). Seventy-five patients had LDL-C above 1.8 mmol/L, and sixty-one had LDL-C above 2.6 mmol/L. Seven patients (8.2%) had LDL-C greater than 4.9 mmol/L.

**Fig. 3:**
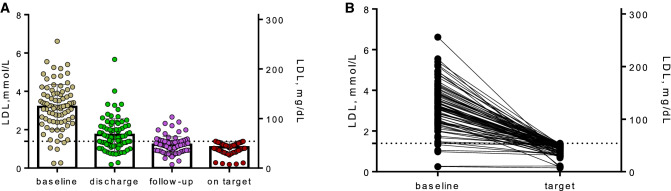
**A** LDL-C levels at baseline, discharge, first follow-up, and “on target” are presented as individual data points. **B** LDL-C levels on baseline and “on target” are shown as individual changes

**Fig. 4:**
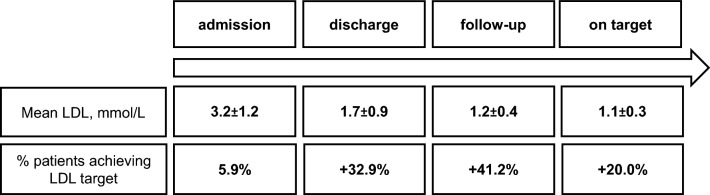
Graphical visualization of LDL-C target (< 1.4 mmol/L) attainment. *BA* bempedoic acid, *PCSK9 mab* PCSK9 monoclonal antibody. To convert mmol/L to mg/dL, multiply by 38.66976. Data are shown as mean and SD

Early initiation of combined lipid-lowering therapy reduced LDL-C at discharge to 1.7 ± 0.8 mmol/L (65.7 ± 34.8 mg/dL). A total of 33 patients (38.8%) were on LDL-C target at discharge of the index hospitalization. On the first post-discharge follow-up, LDL-C levels were on average 1.2 ± 0.4 mmol/L (46.4 ± 15.5 mg/dL), and another 35 patients (41.2%) attained the recommended LDL-C targets, so that a total of 68 patients (80%) reached the LDL-C goal on generically available combination therapy. During ambulatory follow-ups in our lipid clinic all patients who were not “on target” were optimized in regard to lipid-lowering therapy. In one patient we addressed compliance issues, two patients were switched from atorvastatin 80 mg to rosuvastatin 40 mg, in eight patients combined lipid lowering with atorvastatin 80 mg and ezetimibe 10 mg was escalated with bempedoic acid (additional 15% LDL-C reduction), and in six patients with PCSK9 inhibitors (additional 68% LDL-C reduction). In four patients, LDL-C target was attained in the hospital, but was greater than 1.4 mmol/L during follow-up. In these patients, we addressed compliance issues and side-effects, or/and corrected therapy; thereafter, LDL-C target could be re-attained. After therapy escalation, all patients reached LDL-C target levels of 1.4 or below (Figs. 3, 4). Mean LDL-C was 1.1 ± 0.3 mmol/L (42.5 ± 11.6 mg/dL). The overall LDL-C change from baseline was − 60.1 ± 19.2%; the average LDL-C change from baseline was 2.1 mmol/L (81.2 mg/dL), with a substantial inter-individual variability (Supplementary Fig. S1). During the follow-up period, lipid-lowering therapy could be reduced in some patients. Thus, during the final analysis, seven patients received atorvastatin 40 mg and four patients were treated with rosuvastatin 20 mg. All other patients were treated with maximally tolerated statin therapy (atorvastatin 80 mg).

In one patient, we detected a mutation in the LDL-C-receptor gene. The initial LDL-C of this patient was 6.62 mmol/L. On atorvastatin 80/ezetimibe 10 mg LDL-C was reduced to 2.66 mmol/L and after initiation of PCSK9mAb LDL-C was reduced to 0.83 mmol/L.

### Side effects of early combination therapy

Three patients complained of myalgias or abdominal pain early after initiation of atorvastatin 80 mg and ezetimibe 10 mg and refused further participation in the study (Table 2, Fig. 2). During the follow-up period, two other patients complained about muscle pain (2.3%), two about dizziness (2.3%), one about joint pain (1.1%) and one patient had liver enzyme elevation (1.1%). One patient reported dizziness after addition of bempedoic acid.

**Table 2:** Adverse effects. Numbers for both initial cohort (*n* = 88, including three patients who refused further participation) and final cohort (*n* = 85) are shown

Adverse effect	*N* (%) of patients, *N* = 88	*N* (%) of patients, *N* = 85
Muscle pain	4 (4.5%)	2 (2.3%)
Abdominal adverse effects (incl. liver enzyme ↑)	4 (4.5%)	1 (1.1%)
Dizziness	2 (2.3%)	2 (2.3%)
Joint pain	1 (1.1%)	1 (1.1%)

Reason for consent withdrawal in these three patients was adverse effects. Two patients had both muscle pain and abdominal adverse effects

Altogether, side effects were very low and early combined lipid-lowering therapy was well-tolerated. Patients will be followed-up at 6 and 12 months to assess compliance and LDL-C target attainment over time.

## Discussion

In this prospective cohort study, we demonstrate that in high-risk patients after STEMI EAS/ESC-LDL-C targets can be achieved in all patients. Early initiation of combined lipid-lowering therapy with atorvastatin 80 mg and ezetimibe 10 mg on admission resulted in LDL-C target attainment in 80% of the patients at the first follow-up. Escalation with either bempedoic acid or PCSK9 inhibitors reached ESC/EAS-LDL-C targets in all patients. Moreover, reported side effects were rare and combined lipid-lowering therapy was well tolerated.

A large-scale Swedish registry study showed that one out of five patients is at risk of a subsequent cardiovascular event following the first 365 days after an initial myocardial infarction, indicating the risk of this particular patient population [14]. The Improved Reduction of Outcomes: Vytorin Efficacy International Trial (IMPROVE-IT) was the first randomized controlled trial to demonstrate the reduction of atherosclerotic cardiovascular disease (ASCVD) outcomes when ezetimibe was combined with a statin in very high-risk patients after an ACS [15]. The Further Cardiovascular Outcomes Research with proprotein convertase subtilisin/kexin type 9 (PCSK9) Inhibition in Patients with Elevated Risk (FOURIER) trial showed safety and efficacy of adding evolocumab to a statin in ASCVD patients with LDL-C levels > 70 mg/dL on optimized lipid-lowering therapy [16]. ODYSSEY Outcomes: Evaluation of Cardiovascular Outcomes After an Acute Coronary Syndrome During Treatment With Alirocumab (ODYSSEY Outcomes) showed that targeting LDL-C levels to 25–50 mg/dL with alirocumab reduced the number of ACSVD events by 15% in patients with a recent acute coronary syndrome [17]. These studies validated the fact that lowering LDL-C is fundamental to preventing ASCVD events and based on these findings the ESC and the EAS defined new LDL-C targets [2].

According to current guidelines, statins are first-line lipid-lowering therapy for ASCVD risk reduction [2]. Ezetimibe and PCSK9 inhibitors should be initiated only when LDL-C targets are not achieved at follow-up. Optimal responses to moderate and high-intensity statins are LDL-C reductions of 30–40% and > 50%, respectively [2]. However, patients with high cholesterol absorption (so-called “hyperabsorbers”) are characterized by a low-to-normal cholesterol synthesis. These patients exhibit poor response to statins, which explains a high inter-individual variability of LDL-C reductions [9, 18, 19]. Most importantly, in “4 S”, patients with high cholesterol absorption did not benefit from simvastatin treatment [20]. In fact, in this landmark study, high cholesterol absorbers exhibited an increase in cardiovascular events on simvastatin treatment [21]. On the other hand, the Heart Institute of Japan-Proper level of lipid Lowering with Pitavastin and Ezetimibe in acute CoRonary syndrome (HIJ-PROPER) trial showed that only patients characterized as hyperabsorbers exhibited reduced cardiovascular outcomes when treated with ezetimibe on top of statins [22]. These two large-scale lipid-lowering trials demonstrate that individual differences in cholesterol metabolism, impact the efficacy of lipid-lowering therapies not only in terms of LDL-C reductions but also regarding hard cardiovascular outcomes [9]. Finally, a newly published randomized study showed that the statin and ezetimibe combination is non-inferior to a high-dose statin monotherapy in terms of efficacy and safety and patients on combination therapy were more likely to achieve LDL-C levels < 70 mg/dL (< 1.8 mmol/L) [23]. Just recently, a study, including stroke patients, demonstrated that combination of a statin and ezetimibe targeting < 70 mg/dL reduced the risk of subsequent stroke compared to higher target levels [24].

Moreover, evidence from large-scale genetic studies shows that genes that affect dietary cholesterol absorption, such as NPC1L1 (Niemann–Pick C1-Like 1) and ABCG5/8 (ATP-binding cassette transporters G5 and G8), have a greater impact on cardiovascular risk than genes that increase only endogenous cholesterol synthesis such as LDL-C-R (LDL-C-receptor), apoB (apolipoprotein B), HMG (3-hydroxy-3-methylglutaryl coenzyme) reductase and PCSK9 [25–27]. On the background of this evidence, early initiation of combined lipid-lowering therapy with a high-intensity statin and ezetimibe appears to be a logical therapeutic strategy [9, 11–13, 28].

“JaZ” has demonstrated that this approach is feasible, effective, and safe. Early initiation of atorvastatin 80 mg and ezetimibe 10 mg, as a generically available combination therapy, reached ESC/EAS-LDL-C targets < 1.4 mmol/L in 80% of all patients at the first follow-up after the index event. Those who failed to achieve LDL-C targets were started on either bempedoic acid or PCSK9 inhibitors and reached LDL-C targets during further follow-ups. Most importantly, the average LDL-C reduction in “JaZ” from baseline to the last follow-up was ~ 2.0 mmol/L. According to Swedish registry data, an early LDL-C reduction of 2.0 mmol/L in patients after an index MI corresponds with a decrease in all-cause mortality by more than 65%, major cardiovascular events by 60%, and myocardial reinfarctions by 60% during 4-year follow-up [5]. These “real-life” outcome data validate the importance of a “hit hard and hit early” approach in this high-risk patient population.

Moreover, early combined lipid lowering with a high-intensity statin and ezetimibe is well-tolerated (Table 2). We believe that the low rates of side-effects are due to a thorough medical education of each patient. This approach is most effective during the hospital stay of the index event. Patient cards “empower” the patient to actively participate in therapy management during follow-up. Thus, several parties need to participate in this process: the interventional cardiologist, lipidologists, general practitioners, and most importantly, patients themselves. High therapy adherence in JaZ during follow-ups further adds to the concept of “patient empowerment.”

Even though escalation of lipid-lowering therapies with statins, ezetimibe, and PCSK9 inhibitors to reach LDL-C targets is recommended in the ESC/EAS guideline, it is not common practice. In Da Vinci, most patients in Europe with ASCVD were on monotherapy with either a moderate-intensity or a high-intensity statin. Combined lipid-lowering therapy with a statin and ezetimibe was used only in 9% of patients, and PCSK9 inhibitors were prescribed in only 1% of cases [6]. Among very high-risk patients, 2019 ESC/EAS guideline-recommended LDL-C targets were achieved in 22% with a high-intensity statin, in 21% of the patients with a combination of a statin and ezetimibe, and 58% of the patients on a statin and PCSK9 inhibitors. Moreover, in a European retrospective study with > 14,000 patients, approximately 80% of patients had LDL-C > 1.8 mmol/L (70 mg/dL) and were only on a moderate or a high-intensity statin without further escalation of therapy [29]. Despite of apparent failure to attain the recommended LDL-C goal, most patients did not have a change or titration in their regimen. Similar data are available from US registries. In the GOULD registry, lipid-lowering therapy was intensified in only 14% of ACSVD patients with LDL-C levels > 1.8 mmol/L [7]. In fact, in this study, statin dosage was increased in only 6.3% of patients, not at goal, combined lipid lowering with ezetimibe was used in 4%, and PCSK9 inhibitors were added in only 2% of patients, not at goal. These studies suggest that the primary reason for LDL-C target failure at present is a lack of escalation of lipid-lowering therapies [29]. Currently, prescription of ezetimibe for elevated LDL-C levels in Germany is below 10% [30, 31]. Early combination of lipid-lowering therapies with patient empowerment and patient cards to pinpoint LDL-C targets will help to overcome these issues and help to reduce cardiovascular risk in high-risk patients. This job has to be done in the hospital.

Data from this prospective cohort study show that early combination therapy with a high-intensity statin at maximal dosage and ezetimibe is feasible, effective, and safe. Therefore, we suggest considering this approach in future lipid-lowering strategies for high-risk patients.

### Study limitations

Our study has several limitations. First, “Jena auf Ziel” is a non-randomized, observational study. Second, here we report LDL-C target attainment in the hospital and shortly after discharge. Future analyses are needed to address compliance and LDL-C target attainment in the long term. Finally, patients received high-intensity statin and ezetimibe combination regardless of baseline LDL-C. Thus, we cannot appreciate how many patients would have potentially achieved their LDL-C target on statin therapy alone or using other combination therapies.

## Conclusions and future considerations

Early combination therapy with a high-intensity statin at maximal dose and ezetimibe reaches LDL-C targets in 80% of patients after STEMI. Escalation with either bempedoic acid or PCSK9 inhibitors potentially gets all patients on ESC/EAS targets. Combined lipid-lowering therapy is well tolerated and has few side effects.

## Supplementary Information

Below is the link to the electronic supplementary material.

Supplementary file1 (DOCX 28 KB)

## Data Availability

Data are available upon reasonable request.
