# Supplementary figures and images for: Intensive lipid-lowering therapy for early achievement of guideline-recommended LDL-cholesterol levels in patients with ST-elevation myocardial infarction (“Jena auf Ziel”)
Source: Clin Res Cardiol. 2023 Jan 5;112(9):1212–9. doi: 10.1007/s00392-022-02147-3 (PMC10449699; doi:10.1007/s00392-022-02147-3)

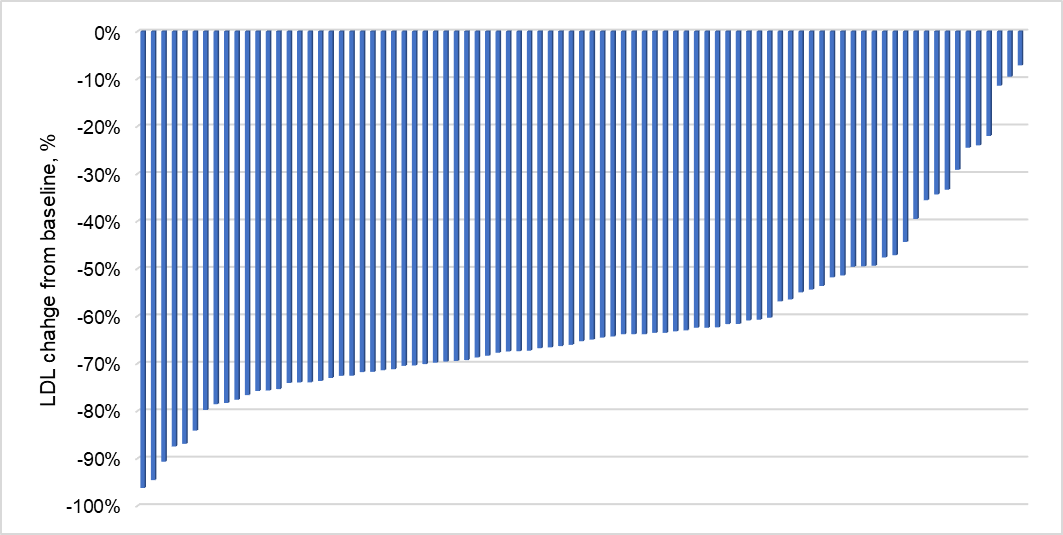
 Supplementary material

Supplementary figure S1. Waterfall plot of LDL change from baseline.

Supplement: Supplementary file 1 — Supplementary file1 (DOCX 28 KB) [file 392_2022_2147_MOESM1_ESM.docx]
